# Transient Alteration of Cellular Redox Buffering before Irradiation Triggers Apoptosis in Head and Neck Carcinoma Stem and Non-Stem Cells

**DOI:** 10.1371/journal.pone.0014558

**Published:** 2011-01-19

**Authors:** Anthony Boivin, Maité Hanot, Céline Malesys, Mira Maalouf, Robert Rousson, Claire Rodriguez-Lafrasse, Dominique Ardail

**Affiliations:** 1 Université de Lyon, Université Lyon-I, Lyon, France; 2 Laboratoire de Radiobiologie Cellulaire et Moléculaire, EA-3738, Faculté de Médecine Lyon-Sud, Oullins, France; 3 Hospices Civils de Lyon, Lyon, France; Universität Heidelberg, Germany

## Abstract

**Background:**

Head and neck squamous cell carcinoma (HNSCC) is an aggressive and recurrent malignancy owing to intrinsic radioresistance and lack of induction of apoptosis. The major focus of this work was to design a transient glutathione depleting strategy during the course of irradiation of HNSCC in order to overcome their radioresistance associated with redox adaptation.

**Methodology/Principal Findings:**

Treatment of SQ20B cells with dimethylfumarate (DMF), a GSH-depleting agent, and L-Buthionine sulfoximine (BSO), an inhibitor of GSH biosynthesis 4 h before a 10 Gy irradiation led to the lowering of the endogenous GSH content to less than 10% of that in control cells and to the triggering of radiation-induced apoptotic cell death. The sequence of biochemical events after GSH depletion and irradiation included ASK-1 followed by JNK activation which resulted in the triggering of the intrinsic apoptotic pathway through Bax translocation to mitochondria.

**Conclusions:**

This transient GSH depletion also triggered radiation-induced cell death in SQ20B stem cells, a key event to overcome locoregional recurrence of HNSCC. Finally, our *in vivo* data highlight the relevance for further clinical trials of endogenous redox modulation to enhance the cytotoxic effects of radiotherapy.

## Introduction

Head and neck squamous cell carcinoma (HNSCC) is among the 10 most common cancers worldwide [1] and, despite new basic and clinical information, the overall 5-year survival rate for HNSCC remains as low as 50% [2]. Along with surgery, radiotherapy plays a key role in the management of early stage and locally advanced HNSCC either alone or, more frequently combined with surgery and/or chemotherapy. In the past few years, a significant 5-year local control and overall survival benefit has been the introduction of concomitant chemoradiotherapy or molecular targeted therapy combined with irradiation [3]. The knowledge about the mechanisms leading to radioresistance and therefore local recurrence of HNSCC has remained very limited up to now. We previously demonstrated a lack of induction of apoptosis in SQ20B cells, a p53 mutated radioresistant HNSCC cell line following either X-ray [4] or carbon ion irradiation [5]. After a transient arrest in G2/M phase following exposure to X-rays or more prolonged after carbon ion, some SQ20B cells undergo mitotic catastrophe whereas the majority of them escape mitotic catastrophe and re-enter the cell cycle. The development of adjuvant therapies in order to force the tumour cells to enter apoptosis after irradiation should therefore be a major improvement towards overcoming the HNSCC resistance to anticancer treatments [6].

Although many types of cancer cells have increased levels of reactive oxygen species (ROS), compared with their normal counterpart [7], [8], which may play an important role in the initiation and progression of cancer [9], [10], excessive levels of ROS can also be toxic to these cells Thus, they become more vulnerable to damage by further ROS insults induced by exogenous agents [11]. Under persistent intrinsic oxidative stress, many cancer cells become well-adapted to such stress and develop an enhanced endogenous antioxidant capacity [12] which makes the malignant cells resistant to exogenous chemical [13] or physical exogenous injuries [14]. The mechanisms of cancer cell redox adaptation may involve multiple pathways to activate redox-sensitive transcription factors such as NF-κB or Nrf-2 [15], [16] which can, among others, lead to the increased expression of anti-oxidant molecules such as SOD, catalase, thioredoxin and the GSH anti-oxidant system [17]. GSH is the major ROS-scavenging system in cells and the important redox modulating enzymes including the peroxidases, peroxiredoxins and thiol reductases rely on the pool of reduced GSH in the cell as their source of reducing equivalents [18]. Therefore, strategies to induce a loss of reduced GSH pool are expected to have a major effect on cell survival and sensitivity toward irradiation by altering the ability of cells to detoxify ROS and therefore by triggering cell death. This can be achieved by targeting its synthesis with buthionine sulfoximine (BSO), an inhibitor of glutamylcysteine ligase (γ-GCL), the rate-limiting enzyme for GSH synthesis. Previous reports have presented evidence for the effectiveness of BSO in inhibiting *in vitro* growth inhibition of cancer cell lines whether used alone [19] or more generally in combination with irradiation [20]–[24] or other drugs [25]–[29]. In contrast to this, only a few results of *in vivo* effects of BSO [25], [30]–[32] have been reported up to now. Moreover, only one phase I clinical trial with BSO and melphalan [33], [34] has been undertaken with disappointing results in terms of GSH depletion. Despite these unfavorable results *in vivo*, a recent review [35] has pointed out the high potential of a redox-modulating strategy to improve cancer treatment.

Epithelial tumors, including HNSCC, contain cellular heterogeneity, some of which, termed cancer stem cells (CSC), possess extensive self-renewal capability and drive tumorigenesis. Up to now, current treatment for HNSCC may selectively kill the differentiated cancer cells, producing tumor regression while sparing the CSC, leading to regrowth and relapse. The recognition of the potential importance of stem cell patterns in tumor renewal has now led to the realization that successful therapies at least need to include CSCs within their range of fatal actions [36], [37].

The aim of this work was to design a therapeutic strategy before irradiation through a transient modification of thiol redox homeostasis in order to overcome radioresistance of HNSCC. This was achieved on SQ20B cells as a model of radioresistant squamous cell carcinoma by the combined use of a GSH depleting agent, dimethylfumarate and the γ-GCL inhibitor BSO before irradiation. Owing to the crucial roles of cancer stem cells in HNSCC tumour initiation, disease recurrence and radioresistance, this pharmacological approach was for the first time tested and validated either in HNSCC stem or non-stem cells. In addition, preclinical successful results are presented which highlight the potential of this redox-modulating strategy in HNSCC treatment.

## Materials and Methods

### Drugs and reagents

Buthionine sulfoximine (BSO), dimethylfumarate (DMF), MTT, SP600125 and other reagents were purchased, from Sigma-Aldrich (Saint-Quentin Fallavier, France). Anti-Bid, anti-JNK (phosphorylated on Thr183/Thr185), anti-ASK-1 (phosphorylated on Thr845) polyclonal antibodies were supplied by Cell Signaling Technology (Danvers, MA), anti-caspase-8 monoclonal antibody from StressGene (Ann Arbor, MI); anti-ASK-1, anti-erk1/2 (phosphorylated on Thr202/Tyr204), anti-p38-MAPK (phosphorylated on Thr180/Tyr182) polyclonal antibodies from Epitomics Inc (Burlingame, CA), anti-cytochrome c monoclonal antibody was supplied by BD Biosciences (San Jose, CA), anti-GAPDH monoclonal antibody from Biodesign International (Saco, ME), anti-Bax polyclonal antibody and the secondary antibodies used (horseradish peroxidase-conjugated goat anti-mouse or anti-rabbit IgG) from SantaCruz Biotechnology Inc (Santa Cruz, CA).

### Cell culture and treatment

The HNSCC SQ20B cells was cultured in Dulbecco's modified Eagle's medium (PAA, Pasching, Austria) supplemented with 10% (v/v) fetal calf serum, 100 unit/ml penicillin, 0.1 g/l streptomycin, 0.04 mg/l hydrocortisone at 37°C in 5% CO_2_.

### Irradiation of cells

Cells were irradiated with X-rays on a Clinac CD irradiator (Varian Medical System, Palo Alto, CA), at a dose of 10 Gy delivered at a dose rate of 3 Gy/min in the Radiotherapy Department of Lyon-Sud Hospital Center.

### MTT assay

About 8.000 cells/well were grown in 96-well microtiter plates, incubated overnight in 100 µl of culture medium and then treated with different concentrations (0 to 500 µM) of DMF and/or BSO for 24 h. 10 µl of MTT labelling reagent (0.5 mg/ml) was added and the cells were incubated for another 4 h at 37°C. The supernatant was removed and 100 µl of 0.04 mol/l hydrochloric acid in isopropanol was added. The absorbance was measured at 595 nm.

### HPLC analysis

Total glutathione was quantified by HPLC analysis. Briefly, proteins were precipitated from the cellular homogenate with sulfosalicylic acid and centrifuged at 13.000×g. The supernatant was then derivatized with *o*-phthalaldehyde. Chromatographic separation was achieved on a 5 µm Spherisorb C18-column, with a mobile phase of methanol – 0.15 M acetate buffer pH 7 (7.5∶92.5). Fluorescence of the glutathione-*o*-phthalaldehyde derivatives was detected at 420 nm with excitation of 340 nm [4].

### Flow cytometry analysis

For cell cycle analysis, cells were pelleted by centrifugation at 300×g, washed once in PBS at 4°C and fixed in ice-cold 70% ethanol before storage at −20°C for at least 24 h until use. After washing, cells were resuspended in 1 ml PBS containing 1 mg/ml RNase-A and 5 µg/ml of propidium iodide and incubated for 20 min in the dark at room temperature before flow-cytometry analysis in red fluorescence (FL 2).

To measure Δψm, cells were incubated for 20 min with 5 µg/ml 5,5′6,6′-tetra-chloro-1,1′,3,3′-tetraethylbenzyl-imidazolcarbocyanine iodide (JC-1) (Molecular Probes, Leiden, The Netherlands) in the dark before flow cytometry analysis in green fluorescence (FL 1).

To measure total caspase activation, cells were incubated with 5 µM CaspACE-FITC-VAD-FMK *in situ* marker (Promega, Madison, WI) for 20 min in the dark. Cells were analyzed by flow cytometry in green fluorescence (FL 1).

Finally, to measure the intracellular ROS level, cells were incubated for 10 min with hydro-ethidium (final concentration 4 µM) in the dark at 37°C. The reaction was stopped at 4°C before analysis by flow cytometry in red fluorescence (FL 2).

### DAPI Staining

Cells were fixed with 4% paraformaldéhyde for 30 min and stained with the fluorescent nuclear dye, 4′,6-Diamidino-2-phenylindole dihydrochloride (DAPI, 5 µg/ml) for 30 min. Apoptotic cells were identified with fluorescence microscopy by the presence of apoptotic bodies.

### Mitochondria isolation

Mitochondria were isolated with the Mitochondria Isolation Kit for Cultured Cells (Pierce, Rockford, IL, USA) according to the manufacturer's instructions. Briefly, 8×10^6^ cells were pelleted by centrifugation at 300×g and included in lysis buffer. After a centrifugation at 12.000×g for 10 min at 4°C, the supernatant containing the cytosolic fraction and the pellet containing the isolated mitochondria were checked for Bax translocation by Western immunoblotting analysis.

### Western immunoblotting

Cells were lysed on ice for 1 h in a buffer containing 150 mM NaCl, 50 mM Tris (pH 8), 1% Triton X-100 and protease inhibitors (protease inhibitors cocktail tablets, Roche, France). Then, lysates were centrifuged for 20 min at 15.000×g. The protein content was determined with the BCA protein assay.

30 µg of total proteins were separated by SDS-PAGE on 14% gels and transferred onto nitrocellulose membrane. The membranes were blocked with TBS-0.1% Tween 20 (TBST) – 5% non-fat dried milk for 1 h, and probed overnight at 4°C with primary antibody (1∶1.000) in TBST – 5% BSA. After washes with TBST, the membrane was probed with HRP-conjugated secondary antibodies diluted at 1∶10.000 in a blocking buffer. After washes, membranes were incubated with enhanced chemiluminescence reagent (Pierce, Rockford, IL, USA), and visualized by Intelligent Dark-Box LAS-3000 (Fujifilm, Tokyo, Japan).

For ASK-1 immunoblot, only 25 µg proteins were separated on 10% reducing gels. For analysis of caspase-8 was the membranes were blocked with PBS-0.05% Tween 20–3% non-fat dried milk. Incubation with primary (1∶2.000) and secondary (1∶7.500) antibodies were performed in the same mixture. Finally, for Bax immune-blot analysis, the primary antibody was diluted at 1∶500 and the secondary at 1∶2000 in TBS-0.05% Tween 20–5% non-fat dried milk.

### SiRNA transfection *in vitro*

The ASK-1-validated siRNA was purchased from Qiagen (Hs_MAP3K5_6 siRNA, Qiagen, Courtaboeuf, France), SQ20B cells were plated in a 6-well plate (5×10^4^ cells/well) 24 h before transfection. Transfection was performed using HiPerfect transfection reagent (Qiagen) according to the manufacturer's instructions, with final siRNA concentration at 150 nM. 48 h after transfection, total cellular ASK-1 was measured by western immunoblotting.

### Cancer stem cells (CSC) isolation

In a first step, SQ20B cells were grown in DMEM supplemented with 25% (v/v) F-12, 100 units/ml penicillin, 0.1 g/l streptomycin, 0.04 mg/l hydrocortisone and 20 ng/ml EGF and 5 µg/ml insulin in order check their ability to form tumourispheres. Then, CSCs were isolated by two successive cell sortings on a FACS VANTAGE SE DIVA (BD Biosciences, San Jose, CA) as described [38]. Briefly, SQ20B CSCs were isolated on their Hoechst dye-excluding property and the expression of the cell surface marker CD44. The Hoechst dye-excluding cells were sorted out from 30×10^6^ SQ20B cells as a side-population (SP) which was further cultured in DMEM at 37°C in 5% CO_2_. This culture medium was supplemented with 5% (v/v) fetal calf serum, 25% (v/v) F-12, 100 units/ml penicillin, 0.1 g/l streptomycin, 0.04 mg/l hydrocortisone and 20 ng/ml EGF. After 10 days of culture, a second cell sorting was performed on a 30×10^6^ SP cells after cell labelling with an antibody to CD44 coupled to FITC (FITC Mouse Anti-human CD44, BD biosciences). The CD44^+^ cells were grown in the same environment as SP cells. The CD44^−^ cells were grown in SQ20B culture medium. After 10 days, cells were trypsinized and used for different experiments.

### *In vivo* treatments

All animal procedures were performed according to local guidelines on animal care. All the details of this study were approved by the CECCAPP, an ethics committee. A suspension of 2×10^6^ SQ20B cells in 150 µl of PBS was subcutaneously inoculated in the right flank region of 5-week-old female athymic nude mice (Harlan France, Gannat, France) under ketamine/rompum anesthesia. When tumours reached 0.3–0.4 cm^3^ in volume, mice were randomly selected for treatment. 32 mg/kg of DMF and 4 mg/kg of BSO were injected in the tumour 4 hours before irradiation (4 Gy/day) for 5 days. Local tumour irradiation was performed under anesthesia at a dose rate of 3 Gy/min. Experiments consisted of 4 groups of 9 mice: PBS, DMF/BSO, 5×4 Gy and DMF/BSO + 5×4 Gy. Clinical observations were carried out daily alongside weekly measurements of body weight and tumour volume, which was calculated according to the formula: 0.5236(LxW^2^) where L and W are, respectively, the length and width diameters.

Total intra-tumoral glutathione was quantified from tumour extracts by HPLC, as described above.

### TUNEL assay

Terminal nucleotidyl transferase-mediated nick end labelling staining was carried out on formalin-fixed, paraffin-embedded tissue sections (5 µm) with the DeadEnd Fluorimetric TUNEL System (Promega, Madison, WI) according to the manufacturer's instructions. Slides were examined using a Zeiss Microscope at a wavelength of 520 nm.

### Statistical analysis

Student's t-test calculation was performed using Microsoft Excel 2003. Student's t-test was used to determine the significance of the differences (a *p-value* of <0.05 was considered statistically significant).

## Results

### Effects of DMF and BSO on cell viability and endogenous GSH depletion

**Figure 1:**
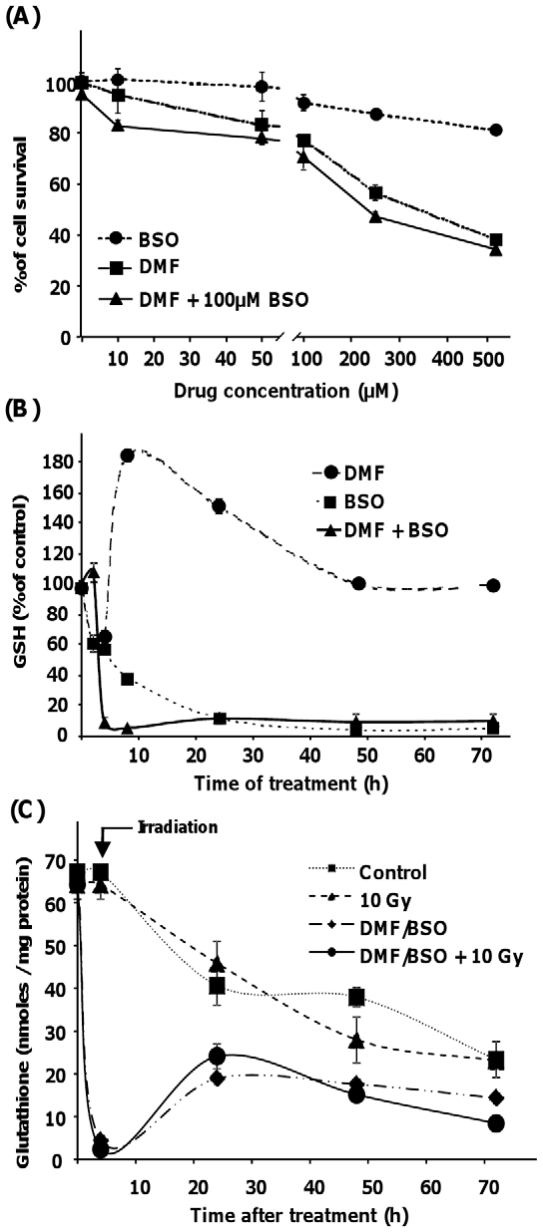
*In vitro* cytotoxicity and efficiency of the glutathione depleting strategy using the pharmacological association of DMF + BSO. SQ20B cells were plated and treated by DMF and/or BSO. Panel A shows the survival of SQ20B cells, determined by the MTT assay, after 24 h of continuous treatment with increasing concentrations of both drugs. Panel B shows the endogenous glutathione level, determined by HPLC, after continuous treatment with DMF (100 µM) and/or BSO (100 µM). In a second set of experiments, panel C shows the endogenous glutathione level with and without 10 Gy irradiation and after treatment with DMF (100 µM) and BSO (100 µM) for 4 h. The drugs were then removed by washing with fresh medium. Results are expressed as mean ± S.D. for three different experiments in triplicate.

In a first set of experiments, we aimed at defining the *in vitro* toxicity of the glutathione-depleting agent DMF and the glutathione biosynthesis inhibitor BSO used either alone or in combination. As shown in Figure 1A, 90% and 83% of cells were still viable 24 hours after a treatment with 100 µM BSO or 100 µM DMF, respectively. Cell viability was considerably reduced at higher concentrations. A combination of 100 µM of each drug for 24 h resulted in a cell viability of around 75%. In order to avoid the cytotoxic effect of drugs, we further examined the effect of a transient pre-treatment of SQ20B cells 4 h before irradiation with 100 µM DMF, 100 µM BSO or in combination (Fig. 1B) which were immediately removed after irradiation by washing with fresh medium. As depicted in Figure 1B, treatment with DMF alone resulted in a rapid and transient decrease in intracellular GSH to about 60% of the initial concentration, 4 h after the treatment, and was followed by a strong and rapid increase in GSH (up to 180% compared to the control) which peaked 8 h after DMF addition. With BSO alone, cells rapidly lost 40% of their endogenous GSH in the first 3 h, followed by a sustained decrease to 10% of the initial concentration at 24 h. When used in combination, DMF and BSO resulted in total cellular GSH depletion at 4 h with complete restoration of the endogenous GSH level at 60 h after washing SQ20B cells with fresh culture medium (Fig. 1C). A 4-hour exposure to DMF and BSO before irradiation was therefore considered as optimal to efficiently deplete SQ20B GSH stores before irradiation without significantly affecting cell viability or subsequent survival in culture.

### Triggering of the intrinsic apoptotic pathway after endogenous glutathione modulation before irradiation

**Figure 2:**
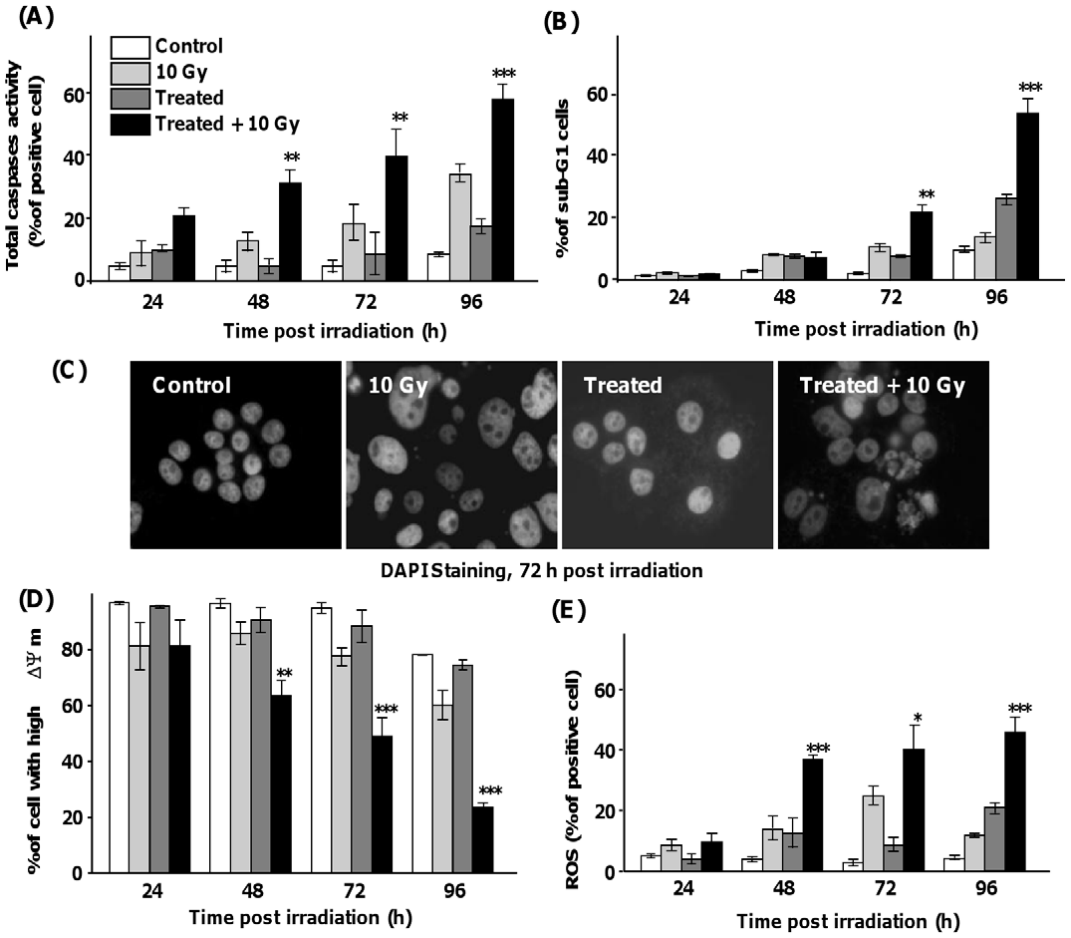
Depletion of endogenous glutathione content before γ-ray exposure triggers radiation-induced intrinsic apoptosis in SQ20B cell line. SQ20B cells were treated with 100 µM DMF and 100 µM BSO for 4 h before irradiation. The drugs were then removed by washing with fresh medium. Total caspase activity and the percentage of cells in the sub-G1 phase were determined by flow cytometry, respectively after VAD-FMK-FITC (A) and propidium iodide (B) staining. Panel C shows the nuclear morphology of cells by DAPI staining 72 h post irradiation. Panels D and E show the mitochondrial alteration after JC-1 staining through the measurement by flow cytometry of the transmembrane potential (A) and after hydro-ethidine staining to measure the reactive oxygen species generated by respiratory chain (B). Results are expressed as mean ± S.D. for three different experiments in triplicate. The statistical significance is expressed as **, p<0.01 and ***, p<0.001 *versus* 10 Gy only.

Figure 2 shows that GSH-depleted SQ20B cells can undergo apoptosis after a 10 Gy irradiation as evidenced by a significant increase in total caspase activity from 48 h after irradiation up to 60% of positive cells 96 h after irradiation (Fig. 2A). To the same extent, up to 60% of pre-treated SQ20B cells were found as hypodiploid (sub-G1) apopototic cells 96 h after irradiation (Fig. 2B). These data were confirmed by DAPI staining which revealed the presence of apoptotic bodies (Fig. 2C) as a result of chromatin condensation and nuclear fragmentation. The involvement of the intrinsic apoptotic pathway was further evidenced by the loss of mitochondrial Δψm and the production of ROS. Irradiation of GSH-depleted SQ20B cells induced a strong decrease in Δψm (Fig. 2D) with a parallel significant increase in ROS production (Fig. 2E) from 48 h after irradiation.

### Effects of transient GSH depletion combined with irradiation on the activation of SAPK pathways and Bax translocation to mitochondria

**Figure 3:**
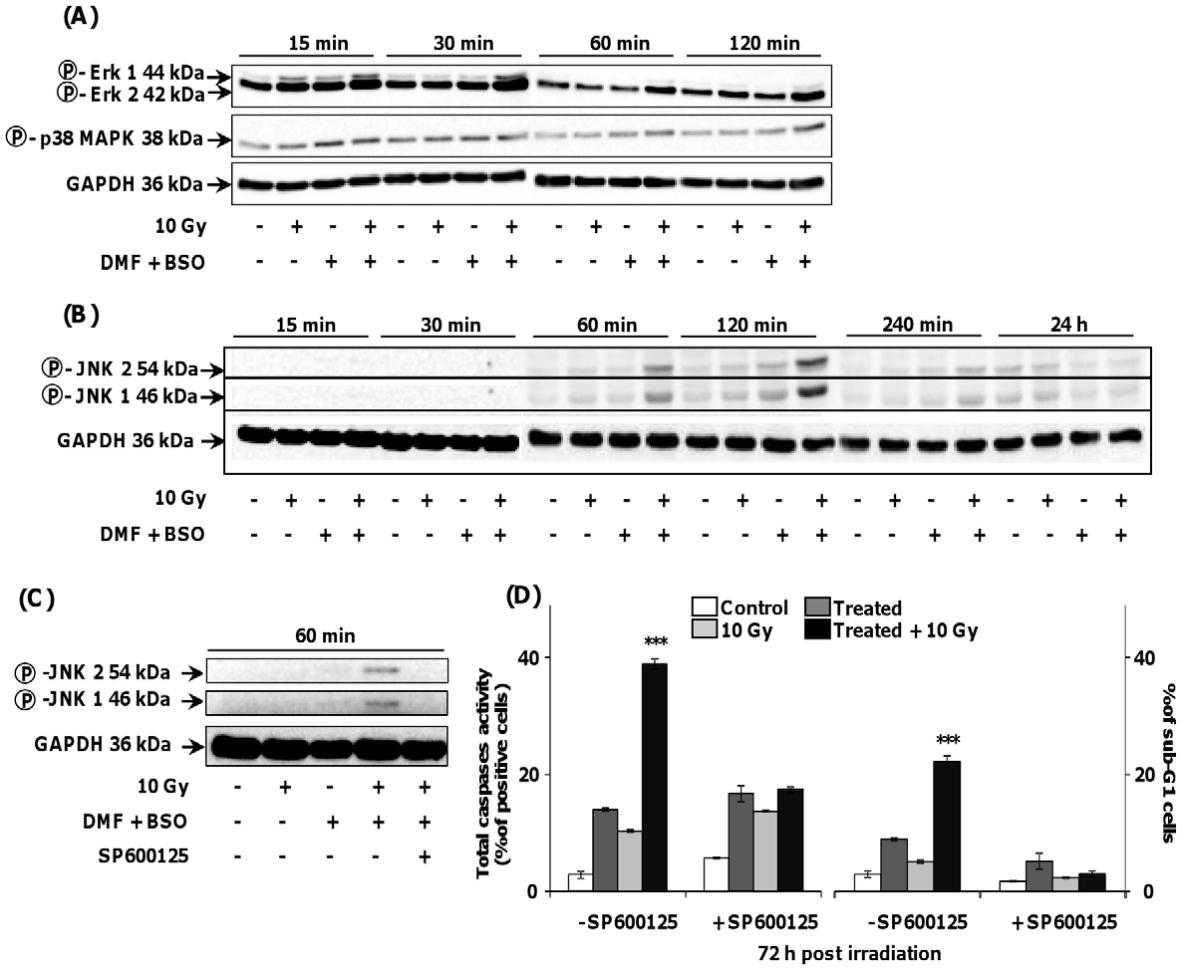
Involvement of the MAPK pathway in the triggering of apoptosis after transient intracellular GSH depletion before irradiation. SQ20B cells were treated with 100 µM DMF and 100 µM BSO for 4 h whereas 10 µM SP600125, a specific JNK inhibitor, was added 1 h before irradiation in the cell culture medium before irradiation. The drugs were then removed by washing with fresh medium. After different points in time after irradiation, cells were harvested and the extracted proteins submitted to Western blot analysis. Panel A: Western blot analysis of phosphorylated Erk, p38 MAPK, and GAPDH. Panel B: Western blot analysis of inhibition of JNK phosphorylation by SP600125. Panel C: Western blot analysis of phosphorylated JNK and GAPDH. Panel D shows the consequence of JNK inhibition in terms of apoptosis estimated by flow cytometry through the total caspase activity (left) and the % of cells in sub-G1 phase (right) measurement 72 h after irradiation. Results are expressed as mean ± S.D. for three different experiments. The statistical significance is expressed as ***, p<0.001 *versus* 10 Gy only.

**Figure 4:**
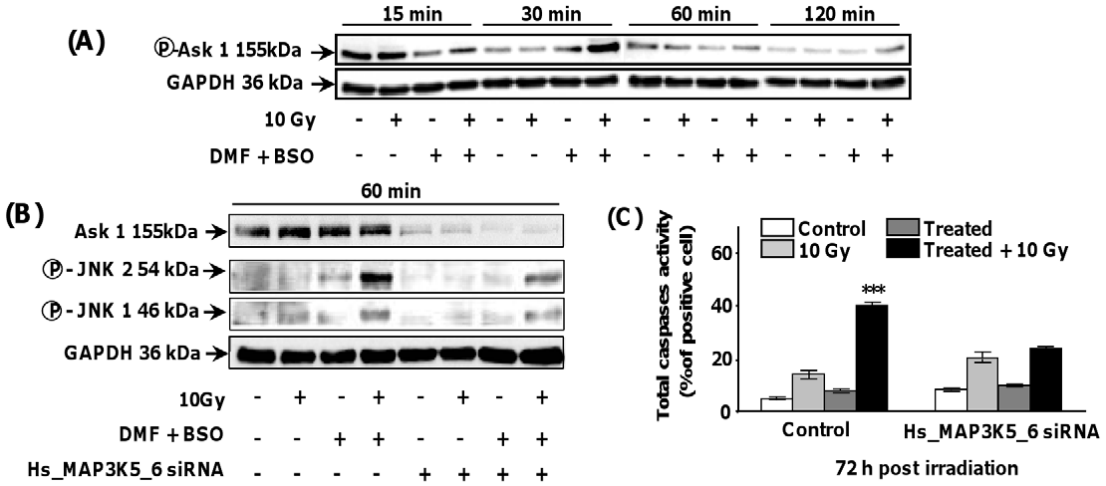
Activation of Ask1 upstream of the MAPK pathway in irradiated GSH-depleted SQ20B cells. SQ20B cells were treated with 100 µM DMF and 100 µM BSO for 4 h before irradiation. The drugs were then removed by washing with fresh medium. At different times post irradiation, cells were harvested and the extracted proteins submitted to Western blot analysis. Panel A: levels of phosphorylated Ask1 Panel B: levels of ASK-1 after specific siRNA transfection and downstream phosphorylation of JNK. Panel C: control of apoptosis through the measurement of total caspase activity 72 h after transfection of irradiated GSH-depleted cells with Ask1 SiRNA. Results are expressed as mean ± S.D. for three different experiments. The statistical significance is expressed as ***, p<0.001 *versus* 10 Gy only.

**Figure 5:**
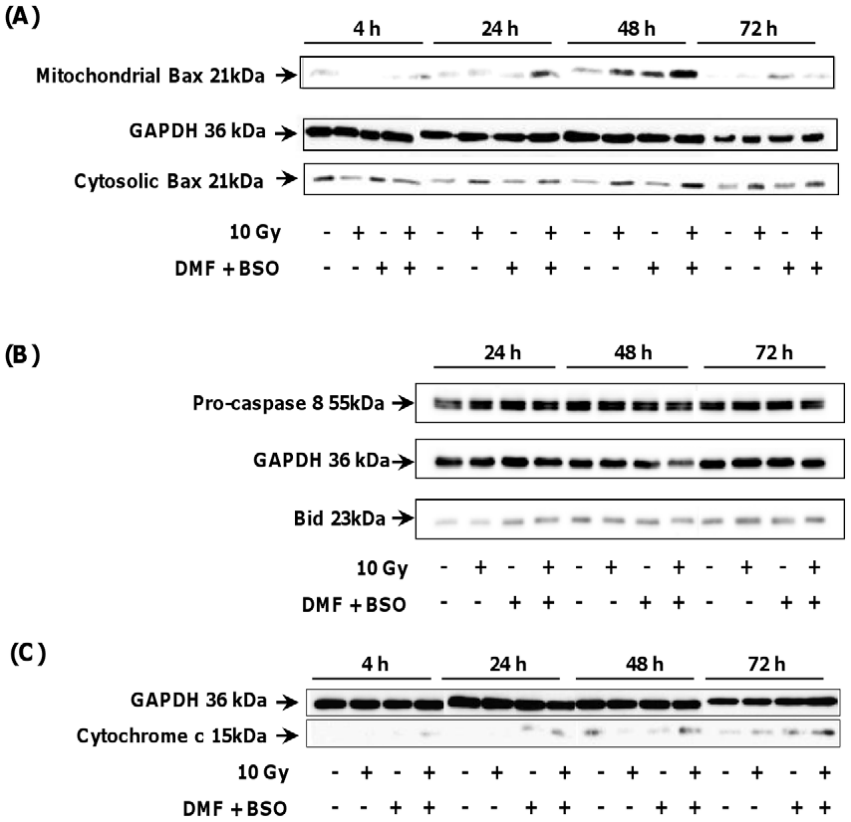
Translocation of the pro-apoptotic protein Bax to mitochondria and release of cytochrome c in the cytosol of irradiated GSH-depleted SQ20B cells. SQ20B cells were treated with 100 µM DMF and 100 µM BSO for 4 h before irradiation. The drugs were then removed by washing with fresh medium. At different time post irradiation, mitochondria were isolated with standard fractionation procedure. The translocation of Bax to mitochondria (Panel A) and the release of cytochrome c in cytosol (Panel C) were measured by Western immunoblotting assay. In parallel, the activation of pro-caspase 8 and the cleavage of Bid were estimated by Western blot analysis (Panel B).

Figure 3A shows a slight and transient increase of the phosphorylation state of p42- and p44-MAPK (Erk) up to 30 min which then declined 60 min after irradiation of GSH-depleted SQ20B cells. In contrast, the p38-MAPK phosphorylation state did not change during the time course studied. As opposed to p38 and Erk-MAPK, a transient increase of the phosphorylation state of JNK/SAPK was observed from 60 min up to 120 min and then declined after irradiation of GSH-depleted SQ20B cells (Fig. 3B) thus strongly suggesting that JNK/SAPK plays a role in the downstream effects of GSH depletion in irradiated SQ20B cells. This result was confirmed by using SP600125, a specific JNK/SAPK inhibitor (Fig. 3C) which resulted in a more than 80% inhibition of apoptosis of irradiated GSH-depleted cells (Fig. 3D). Upstream of JNK, apoptosis signal-regulating kinase (ASK-1) has been reported [39] as a key MAPKKK connecting oxidative stress and ROS to JNK. Figure 4A shows a significant increase in the phosphorylation of ASK-1, 30 min after irradiation of GSH-depleted SQ20B cells followed by a rapid decrease for longer periods. This result was confirmed after downregulating the expression of ASK-1 with a specific short interfering RNA. As depicted in Figure 4B, the successful silencing of ASK-1 resulted in a strong decrease in JNK/SAPK activation in irradiated GSH-depleted SQ20B cells. As a consequence, the number of apoptotic cells was strongly inhibited (Fig. 4C) as evidenced by the measurement of the total caspase activity. The involvement of the intrinsic apoptotic pathway after irradiation of GSH-depleted SQ20B cells was further confirmed by the translocation of the pro-apoptotic Bax protein from the cytosol to mitochondria (Fig. 5A) whereas no cleavage of either pro-caspase-8 and Bid occurred under the same experimental conditions (Fig5B). Additionally, Bax translocation to mitochondria was correlated with the release of cytochrome c in the cytosol, as depicted in Figure 5C.

### Modulation of redox status in HNSCC stem cells as a useful tool to overcome radioresistance *in vitro*?

**Figure 6:**
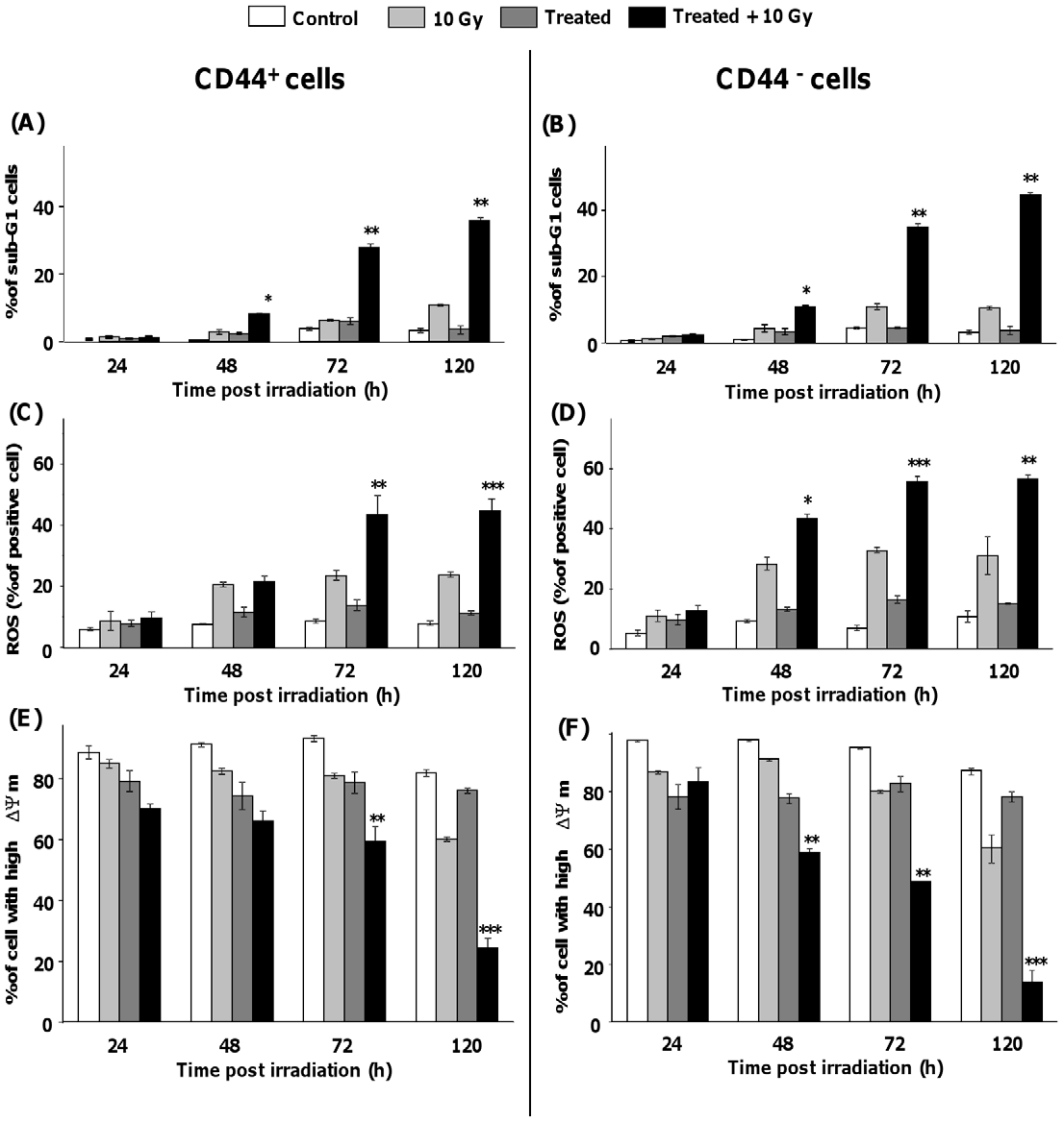
Increase of intrinsic apoptosis in irradiated HNSCC cancer stem cells. After cell sorting, two sub-populations were obtained from the SQ20B carcinoma cell line: CD44^+^ cancer stems cells (A, C, E) and a CD44^−^ side population (B, D, F) which were grown in the SQ20B culture medium for 10 days. Both cell populations were treated with 100 µM DMF and 100 µM BSO for 4 h before irradiation and drugs were then removed by washing with fresh medium. At different times after irradiation, the percentage of apoptotic cells was quantified by flow cytometry analysis. Panels A and B: quantification of apoptotic cells in the sub-G1 phase after propidium iodide staining of CD44^+^ (A) and CD44^−^ (B) sub-populations. Panels C and D: quantification of ROS after hydro-ethidium staining of CD44^+^ (C) and CD44^−^ (D) sub-populations. Panels E and F: Quantification of the mitochondrial transmembrane potential decrease after JC-1 staining of CD44^+^ (E) and CD44^−^ (F) subpopulations. Results are expressed as mean ± S.D. for three different experiments. The statistical significance is expressed as *, p<0.05, **, p<0.01 and ***, p<0.001 *versus* 10 Gy only.

Tumorigenic HNSCC stem cells were isolated from the SQ20B cell line [38] according firstly to their ability for three dimensional growth (tumourisphere formation) and secondly to their Hoechst dye excluding property and the presence of the surface stem cell marker CD44. The fraction of high expressing CD44^+^ subpopulation was found to be as low as 0.1% in the SQ20B cell line. The quantification of the endogenous reduced glutathione content in CD44^+^ and CD44^−^ cells showed that they exhibited approximately the same amount of GSH i.e 76.3±1.6 nmol/mg protein and 73.4±0.9 nmol/mg protein in CD44^+^ and CD44^−^ cells, respectively. Treatment of CD44^+^ and CD44^−^ cells with the association DMF + BSO resulted in a GSH depletion at 4 h to 11% and 17% of basal level in control CD44^+^ and CD44^−^ cells, respectively (data not shown). As depicted in Figure 6A and 6B, GSH-depleted CD44^+^ and CD44^−^ cells were able to undergo apoptosis after a 10 Gy irradiation: the number of cells in the sub-G1 phase increased with time from 48 h after irradiation and show a similar pattern in both subpopulations (up to 40% of sub-G1 cells, 120 h after irradiation). As reported for the whole SQ20B cells, an increase in the level of ROS (Fig. 6C and 6D) and a decrease in the Δψm (Fig. 6E and 6F) occurred in both GSH-depleted subpopulations. All these results demonstrate that our targeted treatment can overcome radioresistance in HNSCC stem cells.

### Modulation of HNSCC redox status as a useful tool to prevent *in vivo* tumor regrowth and relapse?

**Figure 7:**
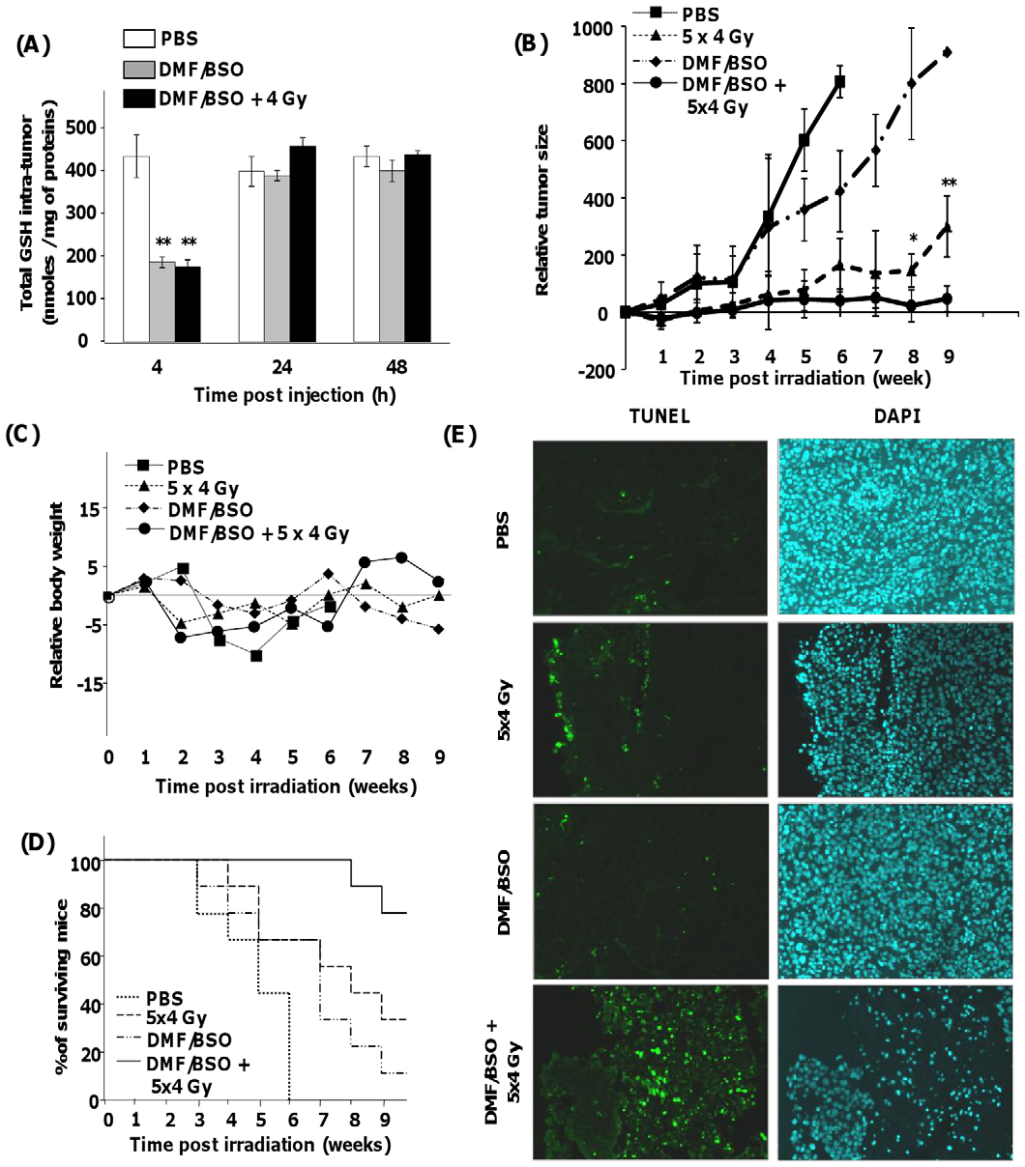
Combined treatment of DMF + BSO with irradiation enhances the survival of mice and inhibits tumor growth without apparent cytoxicity. Panel A shows the efficiency of a single intratumoural injection of 32 mg/kg DMF and 8 mg/kg BSO on the depletion of GSH within the tumour. Panel B shows the relative development of tumour size after combined DMF + BSO treatment each day whether or not associated with an irradiation dose of 20 Gy (4 Gy×5 days). The statistical significance is expressed as *, p<0.05, **, p<0.01 between treated and irradiated tumours versus irradiated tumours. Panel C shows the body weight monitoring of mice after the combined DMF + BSO treatment whether or not associated with an irradiation dose of 20 Gy (4 Gy×5 days). Panel D shows the Kaplan-Meyer survival curves representing the percentage of mice alive at the indicated points in time for each group of the experiment. Panel E shows the detection of apoptosis by TUNEL staining on paraffin-embedded tumor sections.

Preliminary experiments were conducted in order to define the optimal conditions *in vivo* for intra-tumoral GSH depletion. As depicted in Figure 7A, a single DMF (32 mg/kg) and BSO (4 mg/kg) intra-tumoural injection resulted in a 60% decrease in GSH within the tumour 4 h after treatment followed by a total recovery of GSH 24 h after the treatment. Although a progressive increase in tumour volume (Figure 7B) was observed in the control groups, a 4 Gy irradiation during five consecutive days stabilized the development of the tumour up to 5 weeks which then started to develop again. Although a single injection of DMF/BSO had no significant effect on tumour growth compared to the control group, the combination of our GSH-depleting strategy 4 hours before irradiation (one injection before each session of irradiation, 4 Gy for 5 days) drastically enhanced the sensitivity of the xenografted SQ20B tumours to irradiation. At the end of the treatment (week 9), the mean tumour volume had decreased by 95% compared to the control group and by 75% compared to the irradiated control group. This was obtained in the absence of apparent toxicity as evidenced by the recording of mice body weight (Figure 7C). Finally, an increase in the overall survival of mice, as represented by the Kaplan-Meyer curves (Fig. 7D) confirmed the efficiency of the treatment prior to irradiation. TUNEL staining of tumour sections confirmed the results obtained *in vitro:* the combined treatment of DMF/BSO and radiation resulted in large areas of apoptosis (Fig. 7E).

## Discussion

The survival rate of patients with HNSCC has not improved significantly despite multimodal therapy including surgery, radiation therapy and chemotherapy. As up-regulation of antioxidant capacity is a crucial point in the development of radio and/or chemoresistance in numerous cancer cells [40], the aim of our work was to elaborate an experimental protocol relying on the transient depletion, just before irradiation, of intracellular stores of GSH in the radioresistant HNSCC cell line in order to maximize the oxidative stress generated after exposure to γ-rays.

We first experimented the use of dimethylfumarate (DMF) alone to achieve a rapid depletion of the intracellular stores of GSH in SQ20B cells. DMF has been widely used in the treatment of psoriasis [41] and used in a few studies to enhance cytotoxicity of antitumour agents [42] or induce cell cycle arrest in colon carcinoma cells [43]. Apparently, the depleting effect of DMF results from the formation of a conjugate between DMF and GSH that is exported and/or metabolized by the cell [44]. Our results show that DMF alone triggers a rapid depletion of GSH to only 60% of the initial content followed by an overshoot prior to a recovery of the initial GSH concentration 48 h after the beginning of the treatment. This confirms the assumption mentioned above of a redox adaptation of SQ20B cells to oxidative stress through the stimulation of endogenous GSH biosynthesis, once a threshold of low endogenous concentration was reached. In that sense, RT-qPCR analysis showed significant over-expression in SQ20B cells of critical GSH metabolism enzymes such as γ-GCL, glutathione synthase (GS) and gluthathione reductase (GR) when compared to the radiosensitive HNSCC SCC61 cell line (data not shown). We therefore included BSO, an inhibitor of GSH synthesis, before irradiation in order to amplify GSH depletion just before irradiation and to avoid its rapid re-synthesis in SQ20B cells. This experimental approach allowed us to trigger apoptosis in the radioresistant SQ20B cell line (see Figure 2). Previous studies have already tested this pharmacological association in irradiated normal [45] and cancer cells [46], [47] with either toxic effects or only a slight radiosensitizing effect *in vitro*. As a consequence of irradiation, the major oxidative stress obtained under our experimental conditions rapidly activated ASK-1, a ROS-dependent activated kinase [48], [49] which is known to further activate JNK or p38 pathways [50], [51]. Previous work in our laboratory has demonstrated that radioresistance of SQ20B cells is partially correlated to a defect in raft membrane clustering [14]. Overcoming the endogenous antioxidant defences through either exogenous H_2_O_2_ or BSO treatment was shown to restore the activation of an A-Smase followed by its translocation to the outer leaflet of SQ20B cells membrane rafts thereby resulting in raft coalescence into signalling platforms and ceramide release. As ceramide was recently reported to possibly activate ASK-1 [52], the early ceramide release within rafts mentioned above could also act upstream of ASK-1 activation in parallel to that caused by ROS. Once the JNK pathway was activated, the intrinsic apoptotic pathway was triggered through the translocation of the pro-apoptotic protein Bax to mitochondria which resulted in the alteration of this organelle as recently reviewed by Dhanasekaran and Reddy [53]. The great time interval between GSH depletion together with irradiation and triggering of apoptosis reflects the time needed by the cell for DNA double-strand breaks (DSBs) repair after irradiation, the residual DSBs being crucial downstream apoptosis-triggering lesions [54]. As radioresistant cells including HNSCC are very effective in repairing DNA DSBs [55], combining DMF/BSO treatment with radiation was the only way in this study to trigger cell death. As this combination has proven to be also effective with CD44+ cells, this is expected to be a promising strategy to overcome local recurrence in HNSCC.

As epithelial tumors, including HNSCC were recently demonstrated to contain cellular heterogeneity, some of which represent highly tumorigenic subpopulations called “cancer stem-like cells” [38], [56], [57], more effective therapies targeted against this critical population that can overcome radioresistance and improve patient outcome are needed. The second part of our work has therefore been devoted to the validation of our transient GSH-depleting strategy on HNSCC cancer stem-like cells before irradiation. Preliminary determination of the endogenous GSH content shows no significant differences in both CD44^+^ and CD44^−^ subpopulations. Correlatively, RT-qPCR analysis of critical GSH metabolism gene expression showed a significant over-expression of GS and GR in CD44^+^-enriched cells compared to CD44^−^ (data not shown). Similar results have recently been reported for γ-GCL and GS in human breast CSCs [58]. Although the over-expression of such genes in CSC is believed to be involved in the rapid GSH re-synthesis after major oxidative stress or pharmacological depletion (see our results with DMF alone in Figure 1B for example), future work is needed to explain the apparent discrepancy between the endogenous GSH content and the intrinsic radiosensitivity of our subpopulations as previously reported for other cell lines [59], [60]. In all events, exposure of CD44^+^ cancer stem-like cells to irradiation after a 4-hour DMF + BSO treatment resulted in a triggering of the intrinsic apoptotic pathway as efficiently as in the whole SQ20B cell population.

We next evaluated *in vivo* the potential radiosensitizing effect of DMF + BSO on SQ20B tumours. We firstly verified the effectiveness of a single intra-tumoural injection of both drugs in depleting GSH which decreased to 40% of the initial value in the tumour (with or without irradiation) and returned to control values 24 h after the injection of drugs. After delivering a dose of 4 Gy daily for 5 days (20 Gy total), our data show a real efficiency of our pre-treatment illustrated by a decrease of 95% in the mean tumour volume in the absence of apparent toxicity in mice. Moreover, this work clearly shows that the induction of apoptosis is a critical determining factor in tumour radiosensitivity. Finally, 80% of mice were shown to survive up to 9 weeks after irradiation as depicted in the Kaplan-Meyer curves (Figure 7). Taken altogether, our results strongly underline the therapeutic potential of this adjuvant therapy to irradiation in head and neck carcinoma.

As indicated in a very recent review [35], redox adaptation, through an increased intracellular antioxidant capacity, is an important concept that explains to a large degree the mechanisms by which cancer cells, particularly CSCs, become resistant to radiotherapy and/or anticancer agents.

HNSCC is a complex and aggressive cancer characterized by the emergence of therapy-resistant local and regional recurrences. Apart from the development of new molecular targeted [61], [62] or gene therapies [63], our results, in accordance with Trachootham *et al* [35] highlight the potential of using redox-modulating stategy in combination with radiotherapy to specifically eliminate highly tumourigenic CSCs. Moreover, the triggering of apoptosis instead of mitotic cell death under these experimental conditions is a fundamental result to prevent further potential local recurrence of HNSCC.
